# Real‐time feedback in question‐based collaborative learning

**DOI:** 10.1111/medu.15637

**Published:** 2025-03-06

**Authors:** Tom A. Rayner, Melanie Coulson, Amir H. Sam

## 1 WHAT PROBLEMS WERE ADDRESSED?

We introduced question‐based collaborative learning (QBCL) to empower medical students to constructively align their experiential learning with the learning outcomes of the curriculum, to familiarise them with assessment writing processes and to encourage teamworking. This is achieved with item‐writing sessions, in which students are trained to write single best answer (SBA) questions, work in teams to generate questions based on patients seen on clinical placements and ‘map’ these items to their curriculum learning outcomes. These sessions are followed by formative assessments in which students answer peer‐constructed SBA questions and receive feedback from the faculty.

We identified that although these sessions allowed students to collaborate and share clinical experiences, learning was limited by the lack of real‐time feedback. Previous studies have highlighted the importance of timeliness to learners' acceptance and use of feedback.^1^ We additionally noted that when students sat for the formative assessments, there were clear knowledge gaps for the student cohort in some areas that needed to be addressed.

## 2 WHAT WAS TRIED?

We utilised the Learning Activity Management System (LAMS), an open‐source software platform, allowing the faculty to review student‐written questions and analyse formative assessment performance in real time. This enabled real‐time feedback for both question writing and formative assessments.

During question writing sessions, faculty members reviewed the progress of each group taking part in the session by monitoring LAMS. Students worked together in groups of four to six. Where they felt there were significant knowledge or concept‐based errors with the SBA questions, the faculty members provided immediate, face‐to‐face feedback to correct these deficiencies. Similarly, during formative assessment sessions, facilitators reviewed students' performance in real time for each question using the analytics features of LAMS. This enabled faculty members to deliver immediate and tailored feedback to the students at the end of the formative assessments.

## 3 WHAT LESSONS WERE LEARNED?

With increasing demands on medical curricula, finding novel ways to improve time efficiency whilst also maintaining the quality of clinical education is imperative. Although QBCL delivery requires several faculty members to oversee the process in real time, it may enhance the efficiency of feedback delivery in several ways. The opportunity for a real‐time in‐person discussion between students and the facilitators enabled a time‐efficient way of identifying which groups of students would benefit from immediate feedback. Analysing the responses to formative assessment questions in real time allowed faculty members to allocate the appropriate amount of time for focussed feedback per question, helping to determine the detail to go into when providing feedback on the questions immediately after the assessment. Furthermore, because more time was spent on providing feedback on questions students found more challenging in the assessment, we found the students had more time to ask questions, further promoting effective learning.

Whilst we used QBCL for formative purposes, further studies examining the psychometric properties of the items created during QBCL could determine their utility in summative assessments.

## AUTHOR CONTRIBUTIONS

**Tom A. Rayner:** Conceptualization; methodology; project administration; writing – original draft; writing – review and editing. **Melanie Coulson:** Conceptualization; methodology; writing – original draft; writing – review and editing; project administration. **Amir H. Sam:** Conceptualization; methodology; supervision; writing – original draft; writing – review and editing; project administration.

## Data Availability

Data sharing is not applicable to this article as no datasets were generated or analysed during the current study.
